# Graphene-Based Single Crystal TiO_2_ Composites with Exposed Catalytic Interfaces for Efficient Photocatalytic Degradation

**DOI:** 10.3390/ma19101963

**Published:** 2026-05-10

**Authors:** Yaping He, Zihui Sun, Changhu Zhang, Limei Song, Quan Han

**Affiliations:** 1 School of Chemical Engineering, Xi’an University, Xi’an 710065, China; 2 Xi’an Key Laboratory of Environmental Functional Materials Development and Pollution Control, Xi’an 710065, China; 3 Xi’an Key Laboratory of Food Safety Testing and Risk Assessment, Xi’an 710065, China

**Keywords:** photocatalysis, graphene composite single crystal titanium dioxide (GR–TiO_2_SCs), methylene blue, rhodamine B, methyl orange

## Abstract

Three types of graphene–single crystal titanium dioxide composite (GR–TiO_2_SCs) were prepared using the hydrothermal method, employing TiF_4_ and graphite as raw materials with hydrofluoric acid serving as the morphology-directing agent. The phase composition and morphological features of the resultant composites were systematically characterized by X-ray photoelectron spectroscopy, Fourier transform infrared spectroscopy, Raman spectroscopy, and scanning electron microscopy coupled with energy-dispersive X-ray spectroscopy and X-ray diffraction. These complementary characterization results clearly demonstrate that graphene and TiO_2_ single crystals have been successfully hybridized to form a well-defined heterostructure, rather than a simple physical mixture. Photocatalytic performances were evaluated by monitoring the photodegradation behaviors of methylene blue, rhodamine B, and methyl orange solutions under simulated light irradiation, with real-time concentration variations recorded by UV–visible absorption spectroscopy. The composite sample in which TiO_2_SCs were in situ grown and uniformly anchored onto graphene oxide substrates effectively suppressed the self-stacking and agglomeration of individual crystallites, thus delivering the best photocatalytic response. Increased exposure of the active catalytic interfaces of TiO_2_SCs was found to play a key role in elevating the overall photocatalytic activity. The hierarchical assembly protocol developed in this work provides a feasible pathway for the rational design of functional composites with controllable microstructures and tailored properties, which can be further extended to the development of advanced sensing materials.

## 1. Introduction

With the increasing severity of the global energy shortage and environmental pollution, solar-driven photocatalysis has gradually become a core technology for achieving sustainable environmental remediation and clean energy synthesis [1]. Among various semiconductor photocatalysts, titanium dioxide (TiO_2_) has long been the research focus in this field, mainly due to its excellent photochemical stability, non-toxicity, low cost, and good environmental compatibility [2]. However, two inherent defects of TiO_2_ severely restrict its practical application efficiency and industrialization process: (i) The anatase TiO_2_ has a wide bandgap of 3.2 eV, which limits its light absorption range to the ultraviolet (UV) region (λ < 380 nm), and the utilization rate of solar energy is only less than 5%. (ii) The photogenerated electron–hole pairs of TiO_2_ recombine extremely fast (within 1 ns), resulting in more than 80% of charge carriers being consumed before participating in surface redox reactions, which significantly reduces the photocatalytic quantum efficiency [3].

In view of the above bottlenecks, the construction of graphene–TiO_2_ heterojunctions has been proved to be one of the most effective modification strategies. The key lies in the formation of stable Ti–O–C covalent bonds at the interface of graphene and TiO_2_, which can establish unobstructed charge transfer channels and effectively inhibit nonradiative recombination of photogenerated carriers [4]. Furthermore, the hybridization of graphene can regulate the electronic structure of TiO_2_ through the orbital coupling between C 2p and O 2p, narrow the effective bandgap to 2.75 eV, and extend the photoresponse range to the visible light region, thereby increasing the visible-light utilization efficiency of photocatalytic hydrogen evolution to 78% [5]. In addition, graphene has an ultrahigh room-temperature electron mobility (≈200,000 cm^2^ V^−1^ s^−1^) which enables it to act as an efficient electron acceptor and storage medium, accelerating the separation of photogenerated electron–hole pairs, increasing the number of exposed active sites, and optimizing the surface adsorption–desorption kinetics of reactants, thus synergistically improving the overall photocatalytic performance of TiO_2_ [6,7].

Numerous studies have verified that the rational combination of graphene and TiO_2_ is a versatile strategy to overcome the intrinsic defects of TiO_2_ and achieve a leap in photocatalytic performance [8]. As a two–dimensional layered support material, graphene can effectively inhibit the agglomeration of TiO_2_ nanoparticles, making TiO_2_ disperse uniformly with smaller particle size, thus increasing the specific surface area and exposing more catalytic active sites. At the same time, the large specific surface area of graphene can provide sufficient adsorption sites for pollutant molecules, and it can also act as a photosensitizer to extend the light absorption range of the composite to the visible light region. Relevant studies have shown that the introduction of graphene can redshift the absorption edge of TiO_2_ to the long-wavelength direction, and the carbon vacancies formed spontaneously during the growth process of graphene can further reduce the recombination rate of electron–hole pairs [6].

Typical cases have confirmed the effectiveness of this modification strategy: the TiO_2_–pillared multilayer graphene nanocomposite (T–MLGs) with a TiO_2_ mass fraction of 64.3 wt% achieves a photodegradation efficiency of 78% after 150 min of LED irradiation, and its pseudo-first-order reaction rate constant is 3.89 times that of pure TiO_2_ while maintaining good stability and reusability after five consecutive cycles [7]. Similarly, compared with pure TiO_2_ nanotubes (TNTs), the graphene–TiO_2_ nanotube (TNT) composites have a threefold improvement in photocatalytic activity for malachite green degradation [8]. In addition, the photocatalytic performance of graphene–TiO_2_ composites can be further improved by heteroatom doping modification of graphene. For example, N-doped graphene quantum dots (N–GQDs) can promote the efficient transfer of interfacial charges between graphene and TiO_2_, reducing the effective bandgap to 1.53 eV [9]; boron-doped graphene anchored on the (101) surface of anatase TiO_2_ can form stable B–O covalent bonds, which significantly enhance the chemical activity of the carbon layer and strengthen the interfacial electronic coupling [10].

Collectively, these studies fully indicate that the introduction of graphene can significantly improve the photocatalytic efficiency of TiO_2_ through three aspects: bandgap engineering, accelerated charge separation, and surface activity modulation [8,11]. It is particularly noteworthy that the photocatalytic performance of graphene–TiO_2_ composites has a strong crystal facet dependence: the {100} facet of TiO_2_ shows the best photocatalytic activity due to the formation of stable Ti–C interfacial bonds and efficient charge migration, while the {001} facet has relatively poor activity because of the weak electronic coupling at the heterojunction [11]. This finding lays an important foundation for the crystal facet engineering of graphene–TiO_2_ composites.

Despite the remarkable progress made in the research of graphene–TiO_2_ composites, it is worth noting that most of the existing studies focus on traditional TiO_2_ nanoparticles or nanotube assemblies. In sharp contrast, the rational design and in situ construction of graphene-supported TiO_2_ single-crystal heterostructures with controllable exposed facets and stable Ti–O–C interfacial bonds have not been fully explored, which has become a key research gap in this field as well as the core motivation and innovation direction of this study. Traditional photocatalysis research mainly focuses on nano-sized TiO_2_ particles, which is due to their large specific surface area and abundant surface active sites. However, such nanoscale systems have inherent practical application defects: severe and irreversible particle agglomeration, coupled with difficult post-reaction recovery, which significantly reduces the accessible active surface area and impairs the long-term catalytic stability in continuous practical applications [12]. These bottlenecks urgently require the development of new photocatalytic materials with stable structure and high performance.

In this context, TiO_2_ single crystals have attracted widespread attention as a promising alternative. Compared with TiO_2_ nanoparticles, TiO_2_ single crystals have well-defined surface atomic arrangements, low bulk defect concentrations, and excellent structural stability, which make them ideal model systems for studying the intrinsic photocatalytic mechanism and designing practical photocatalytic devices [13]. Recent studies have further confirmed that the synergistic effect between different crystal facets of TiO_2_—especially the high-surface-energy {001} facets and the thermodynamically stable {101} facets—can form built-in surface heterojunctions, which effectively promote the spatial separation of photogenerated electron–hole pairs and thus significantly improve the photocatalytic activity [14]. This provides a new idea for the design of high-performance TiO_2_-based photocatalysts.

Based on the above analysis, the combination of graphene and TiO_2_ single crystals is expected to construct heterostructures with atomically flat interfaces and directional charge transfer channels, which not only helps to deeply explore the intrinsic catalytic mechanism of graphene–TiO_2_ heterojunctions but also is expected to achieve a breakthrough in photocatalytic performance. However, a comprehensive review of the recent literature shows that the targeted research on graphene–TiO_2_ single-crystal composites is still in the initial stage, especially in the aspects of controlled synthesis, systematic interfacial characterization, and in-depth analysis of the mechanism of performance improvement [15]. At present, there are still relatively few systematic studies in this field, which severely restricts the practical application of graphene–TiO_2_ single-crystal composites.

At present, most of the existing methods for preparing graphene–TiO_2_ single-crystal composites simply extend the conventional methods designed for nanoparticle systems (such as hydrothermal and sol–gel methods) to the combination of graphene and TiO_2_ single crystals, which often fail to accurately control the interfacial microstructure and electronic interaction between graphene and TiO_2_ single crystals. Although chemical vapor deposition (CVD) provides a feasible way for the direct growth of high-quality graphene on TiO_2_ single-crystal substrates, which may form closely contacted van der Waals heterostructures, the systematic study of CVD growth parameters, interfacial coupling mechanisms, and their specific effects on photocatalytic performance is still insufficient and needs in-depth exploration [15]. This further highlights the necessity and innovation of this study.

It is worth emphasizing that micron-sized TiO_2_ single crystals are more suitable for practical applications compared with nano-sized TiO_2_ single crystals, as they integrate easy recoverability (realizing gravity-driven rapid separation) and excellent charge transport performance due to their defect-poor crystal lattices. More importantly, the recent progress in crystal facet engineering has made it possible to controllably expose the high-activity crystal planes of micron-sized TiO_2_ single crystals. For example, the anatase {001} facet has 100% undercoordinated Ti atoms, which can increase the CO_2_ adsorption energy by more than 100% compared with the {101} facet [16]; the synergistic separation of electron–hole pairs occurs between adjacent {001} (reductive) and {101} (oxidative) facets, which reduces the carrier recombination rate by 3–7times [16,17]. This provides a favorable condition for the preparation of high-performance graphene–TiO_2_ single-crystal composites.

The strategic shift from traditional TiO_2_ nanoparticle dispersion to facet-controlled micron-sized TiO_2_ single crystals has dual advantages: on the one hand, it solves the practical application obstacles of nanophotocatalysts (such as agglomeration and difficult recovery); on the other hand, it further improves the intrinsic photocatalytic activity of TiO_2_. Micron-sized TiO_2_ single crystals show excellent photocatalytic performance due to their unique structural advantages and favorable charge dynamics. In practical applications, the rigid micron-sized structure endows them with excellent structural stability, which can effectively prevent agglomeration and structural collapse during repeated catalytic cycles. Therefore, clarifying the synthesis–structure–performance relationship of micron-sized graphene–TiO_2_ single-crystal composites is crucial for the development of high-performance photocatalysts that balance catalytic activity, long-term stability, and recyclability, which is also one of the core objectives of this study.

Accordingly, this study focuses on the in situ growth of TiO_2_ single crystals on graphene substrates using a hydrothermal method, with TiF_4_ and graphite as raw materials and hydrofluoric acid as a morphology-directing agent to controllably prepare graphene–TiO_2_ single-crystal composites (GR–TiO_2_SCs). To optimize the photocatalytic performance of GR–TiO_2_SCs, three different synthetic strategies were adopted to prepare novel GR–TiO_2_SCs photocatalysts, and their photocatalytic activities for the degradation of typical organic dye pollutants were systematically evaluated. Meanwhile, comprehensive characterization techniques were used to systematically study the microstructure, chemical composition, interfacial interaction, and optical properties of the prepared GR–TiO_2_SCs. The core purpose of this study is to establish a high-quality model system for exploring the charge separation and transfer mechanism at the graphene–semiconductor interface and to provide a feasible and innovative strategy for the design and preparation of highly efficient, stable, and recyclable new photocatalysts for environmental remediation. This study is expected to fill the research gap in the field of graphene–TiO_2_ single-crystal composites and promote the practical application of TiO_2_-based photocatalysts in environmental remediation.

## 2. Experimental

### 2.1. Apparatus and Measurements

T1810 UV–visible spectrophotometer: Beijing spectrometer automation technology Co., Ltd. (Beijing, China) was used for the exploration of photocatalytic degradation of methylene blue, Rhodamine B and methyl orange. Scanning electron microscopic (SEM) measurements were carried out on a scanning electron microscope JSM–6700F (JEOL Ltd., Tokyo, Japan) at 15 kV, equipped with an energy-dispersive X-ray spectrometer (EDX) for elemental composition analysis. X-ray diffraction (XRD) observations were performed using a Bruck, Germany, D2PHASER diffractormeter with curved graphite crystal filtered Cu–Kα radiation (λ = 0.15418 nm). The DL–180 ultrasonic cleaning machine (35 KHz, Haitian Elec. Instr. Factory, Ningbo, Zhejiang, China) was used to dissolve and form homogeneous solution. X-ray photoelectron spectroscopy (XPS) measurements were performed on a Thermo Scientific ESCALAB 250Xi spectrometer with Al Kα radiation (Thermo Fisher Scientific, Waltham, MA, USA) to analyze the chemical composition and chemical states of elements. Fourier transform infrared (FTIR) spectra were recorded on a Nicolet iS50 FT–IR spectrometer (Thermo Fisher Scientific, Waltham, MA, USA) in the range of 4000–400 cm^−1^ using the KBr pellet method. All the electrochemical experiments were conducted at room temperature (25 ± 2 °C).

### 2.2. Reagents

Titanium tetrafluoride TiF_4_ was obtained from Tianjin Yingda Sparseness and Noble Reagent Chemical factory (Tianjin, China). Hydrogen fluoride HF was obtained from Luoyang Hao Hua Chemical Reagent Ltd. (Tianjin, China). Concentrated hydrochloric acid was obtained from Chengdu Kelong Chemical Co., Ltd. (Chengdu, China). Methylene blue (MB), methyl orange (MO), and potassium hydroxide were obtained from Sigopharm Chemical Reagent Co., Ltd. (Shanghai, China). All other chemicals were of analytical reagent grade, and doubly distilled water was used in all the experiments. A 0.1 M sodium phosphate-buffered saline (PBS) with various pH values was prepared by mixing stock standard solutions of Na_2_HPO_4_ and KH_2_PO_4_ and adjusting the pH value with 0.1 M H_3_PO_4_ and NaOH.

### 2.3. The Preparation of Graphene–Single-Crystal Titanium Dioxide Composite

#### 2.3.1. The Preparation of Single-Crystal Titanium Dioxide

Single crystals TiO_2_ (TiO_2_SCs) were synthesized with minor adjustments referring to previously reported methods [18,19]. Initially, a hydrochloric acid solution was adjusted to pH 2.1 by adding deionized water. A 0.04 mol/L TiF_4_ solution was then prepared by dissolving an appropriate quantity of TiF_4_ powder in the pH-adjusted hydrochloric acid solution, with gentle stirring to ensure full dissolution. This 0.04 mol/L TiF_4_ solution was further diluted to 5.33 mmol/L using deionized water. Next, the diluted TiF_4_ solution was blended with a 10 wt% HF solution and magnetically stirred for 10 min to get a uniform, colorless, and transparent mixture. This mixture was cautiously transferred into a stainless-steel autoclave equipped with a Teflon liner. The autoclave was tightly sealed and placed in an electric oven, maintained at a steady 180 °C for 20 h to conduct the hydrothermal reaction. After the reaction finished, the autoclave was allowed to cool down to room temperature naturally. The resulting product was alternately washed with anhydrous ethanol and deionized water several times until the washing solution became neutral, which helped eliminate residual impurities and unreacted raw materials. Eventually, the purified product was dispersed in anhydrous ethanol, yielding TiO_2_SCs for further experimental use.

#### 2.3.2. The Preparation of Graphene Oxide and Graphene

Graphene oxide (GO) was prepared from graphite powder by means of a modified Hummers method as reported in the literature [20]. To synthesize graphene (GR), 500 mg of GO was initially added into a 250 mL three-necked flask, with 50 mL of doubly distilled water and 50 mL of absolute ethanol subsequently poured into the flask. The mixed system was thoroughly dispersed, and 4 mL of hydrazine was added afterward, followed by continuous stirring throughout the night to guarantee adequate reduction. Once the reduction reaction was completed, the mixture was subjected to centrifugation. The resulting precipitate was washed repeatedly with distilled water until the pH of the washing solution reached 7.0 rapidly. The product obtained was then vacuum-dried at 60 °C for 24 h, thus obtaining graphene (GR).

#### 2.3.3. The Preparation of Graphene Single-Crystal Titanium Dioxide Composite

(1) Preparation of Graphene-Coated Titanium Dioxide Single-Crystal Composite

An appropriate amount of ultrasonically dispersed graphene oxide (GO) solution (1 mg/mL) was added to a mixed solution containing 40 mL of titanium tetrafluoride (0.533 mmol/L) and 0.533 mL of 10 wt% hydrofluoric acid (HF). The resulting mixture was transferred into a hydrothermal reaction autoclave and reacted at 180 °C for 20 h. After the reaction, the product was centrifuged and repeatedly washed with deionized water until the washing solution reached neutrality. The obtained sample was labeled as TiO_2_SCs–1#.

(2) Preparation of Graphene Grown on Titanium Dioxide Single-Crystal Substrate

An appropriate amount of single-crystal TiO_2_ (TiO_2_SCs) was added to an ultrasonically dispersed GO solution (1 mg/mL). The mixed dispersion was transferred into a 250 mL three-necked flask, followed by the addition of 50 mL of doubly distilled water and 50 mL of absolute ethanol for uniform dispersion. Then, 4 mL of hydrazine was added to the system, and the mixture was continuously stirred overnight to ensure complete reduction. After the reduction reaction, the mixture was centrifuged, and the precipitate was repeatedly washed with distilled water until the pH of the washing solution rapidly reached 7.0. Finally, the product was vacuum-dried at 60 °C for 24 h to obtain the sample, which was labeled as TiO_2_SCs–2#.

(3) Preparation of Titanium Dioxide Single Crystals Grown on Graphene Substrate

An appropriate amount of ultrasonically dispersed GR solution (1 mg/mL) was added to a mixed solution consisting of 40 mL of titanium tetrafluoride (0.533 mmol/L) and 0.533 mL of 10 wt% HF. The mixture was placed into a hydrothermal reaction autoclave and reacted at 180 °C for 20 h. After the hydrothermal reaction, the product was washed with deionized water and centrifuged repeatedly until the washing solution was neutral. Subsequently, the product was freeze-dried in a lyophilizer, and the resulting sample was labeled as TiO_2_SCs–3#.

(4) Calcination of Graphene–Titanium Dioxide Single-Crystal Composites

Samples TiO_2_SCs–1#, TiO_2_SCs–2# and TiO_2_SCs–3# were separately placed in crucibles and calcined in a muffle furnace. The calcination process was carried out with a programmed temperature rise: starting from room temperature (25 °C), the temperature was increased at a rate of 5 °C/min to the target calcination temperature of 450 °C, and this temperature was maintained for 120 min. After the calcination was completed, the samples were naturally cooled to room temperature, ground into a uniform powder, and stored in a sealed container for subsequent use.

#### 2.3.4. Photocatalytic Experiments

(1) Blank Experiments for Photocatalytic Degradation

To rule out the influence of self-degradation of dye solutions under ultraviolet (UV) irradiation, blank experiments were performed for methyl orange (MO), methylene blue (MB), and rhodamine B (RhB). The specific procedure was carried out as follows: 50 mL of each dye solution with a concentration of 10 mg/L was separately placed in a clean beaker. Prior to UV irradiation, all dye solutions were subjected to dark treatment for 24 h to eliminate the effect of light-independent self-degradation. A UV lamp with a wavelength of λ = 365 nm was used as the light source for the blank experiments. During the irradiation process, 4 mL of solution was sampled every 15 min, and a total of 8 samples were collected for each dye. The absorbance of each sampled solution was measured and the variation trend of absorbance of different dyes with irradiation time was plotted to confirm the extent of dye self-degradation.

(2) Photocatalytic Degradation Experiments of GR–TiO_2_SCs

For the photocatalytic degradation experiments of graphene–titanium dioxide single-crystal composites (GR–TiO_2_SCs), 0.01 g of GR–TiO_2_SCs sample was accurately weighed and added to a beaker containing 50 mL of 10 mg/L dye solution (MO, MB, or RhB). The mixture was first placed in a dark environment for 24 h with continuous stirring to ensure sufficient adsorption of dye molecules on the surface of GR–TiO_2_SCs and achieve adsorption–desorption dynamic equilibrium. After the dark adsorption period, 4 mL of the mixture was sampled and labeled as A_1_ (corresponding to the initial state before photocatalytic reaction). Subsequently, the 365 nm UV light source was turned on, and a magnetic stirrer was activated to maintain uniform mixing of the reaction system, thus initiating the photocatalytic degradation experiment. During the reaction, 4 mL of the mixture was sampled every 15 min, and this sampling process was repeated 8 times consecutively. The absorbance of each collected sample was measured using a UV752 UV–Vis spectrophotometer (Beijing spectrometer automation technology Co., Ltd., Beijing, China) after centrifugation to remove the GR–TiO_2_SCs catalyst.

(3) Calculation of Degradation Rate

After each sampling, the collected solution was centrifuged to completely separate the GR–TiO_2_SCs catalyst from the dye solution. An appropriate amount of the centrifuged supernatant was drawn into a cuvette, and its absorbance was measured using a UV–Vis spectrophotometer, which was denoted as *A_t_* (absorbance at a specific reaction time). The degradation rate of the dye was calculated using the following formula:

$$\mathrm{Degradation}\;\mathrm{Rate}=\frac{A_{0}-A_{t}}{A_{0}}$$

where *A*_0_ refers to the absorbance of the dye solution without adding any catalyst (measured under the same experimental conditions), and *A_t_* represents the absorbance of the dye solution at a specific reaction time after adding the GR–TiO_2_SCs catalyst and UV irradiation.

## 3. Results and Discussion

### 3.1. Construction Strategies of Graphene–Single Crystal Titanium Dioxide Composite

**Scheme 1:**
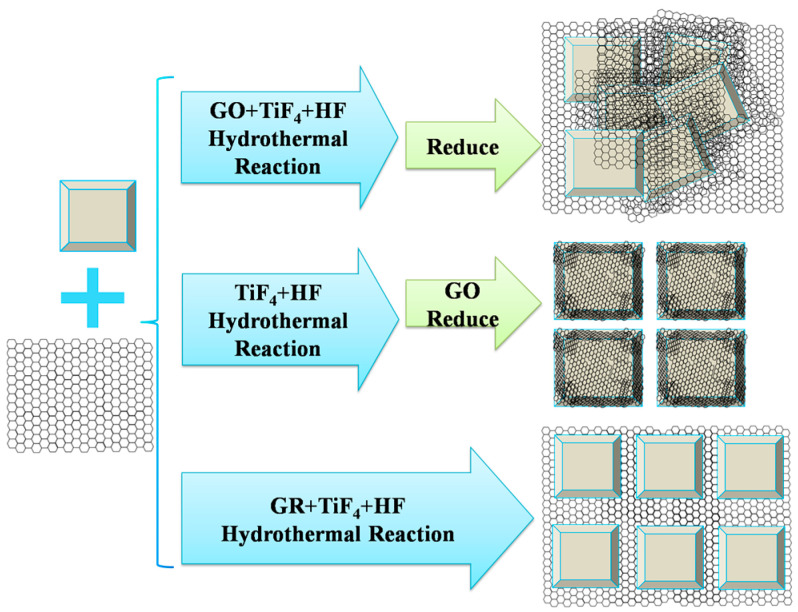
Schematics of graphene–single crystal titanium dioxide composite.

Three distinct synthetic routes were designed to construct GR–TiO_2_SCs with tailored microstructures, as schematically depicted in Scheme 1. (1) Route 1 for TiO_2_SCs–1#, graphene-wrapped TiO_2_SC composite: GO nanosheets were employed as the growth template, and TiO_2_ single crystals were in situ hydrothermally grown on the GO surface via the fluorine-induced crystal growth route using TiF_4_ and HF as precursors. Subsequent chemical reduction of the GO matrix yielded a 3D hierarchical composite with TiO_2_ single crystals tightly wrapped by GR nanosheets. (2) Route 2 for TiO_2_SCs–2#, graphene-decorated TiO_2_SC substrate: Monodisperse TiO_2_ single crystals were first pre-synthesized via a hydrothermal reaction of TiF_4_ and HF. The as-obtained TiO_2_SCs were then mixed with GO nanosheets, followed by in situ reduction of GO to form a composite structure where graphene nanosheets were uniformly grown and anchored on the surface of TiO_2_ single-crystal substrates. (3) Route 3 for TiO_2_SCs–3#, TiO_2_SCs grown on graphene substrate: A one-pot hydrothermal strategy was adopted, where pre-reduced graphene (GR) nanosheets were directly used as the growth substrate. TiO_2_ single crystals were in situ nucleated and epitaxially grown on the GR surface via the reaction of TiF_4_ and HF, resulting in a composite with well-dispersed TiO_2_SCs anchored on the graphene framework.

### 3.2. Characterizations of Surface Morphology and Elemental Distribution

**Figure 1:**
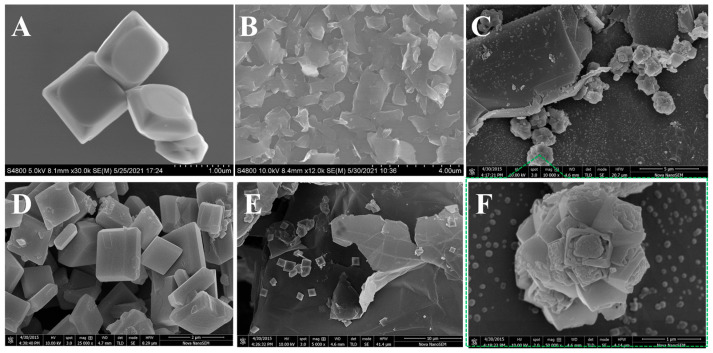
SEM images of TiO_2_SCs. (**A**) GR, (**B**) TiO_2_SCs–1#, (**C**) TiO_2_SCs–2#, (**D**) TiO_2_SCs–3#, (**E**) partial enlargement of TiO_2_SCs–1# (**F**).

As shown in Figure 1A, the TiO_2_ single crystals (TiO_2_SCs) exhibit well-defined cubic morphologies corresponding to anatase phase. These anatase TiO_2_SCs display distinct cubic geometries with relatively smooth surfaces and sharp, well-demarcated edges. The cubic particles are closely packed, with sizes in the micrometer range. Notably, clear T-shaped cubic features are observed, which confirms the successful synthesis of anatase TiO_2_ single crystals with regular and uniform morphological characteristics. 

Figure 1B presents the SEM micrograph of reduced graphene (GR) nanosheets, which exhibit a typical two-dimensional (2D) lamellar structure. The GR sheets appear as discrete, ultra-thin flakes with inherent wrinkling and slight agglomeration. This morphological feature is consistent with the high aspect ratio and weak van der Waals forces between GR layers, which are responsible for its exceptional mechanical strength, electrical conductivity, and structural flexibility.

For TiO_2_SCs 1# (Figure 1C), the cubic TiO_2_SCs with relatively regular morphologies are tightly anchored on the surface of GR nanosheets. The GR sheets maintain their characteristic lamellar structure, while the TiO_2_SCs retain their distinct cubic particle shapes, with mild agglomeration observed (Figure 1F). The intimate attachment between TiO_2_SCs and GR nanosheets can be clearly observed, which implies a strong interfacial interaction between the two components. This interfacial contact is crucial for modulating the physicochemical properties of the composite.

The SEM image of TiO_2_SCs–2# (Figure 1D) reveals two distinct structural components within the field of view: cubic TiO_2_SCs and GR sheets. GR sheets exhibit a translucent and crumpled thin-film structure with uneven thickness, which are dispersed on the surface and periphery of cubic TiO_2_SCs. In local regions, GR sheets closely adhere to the crystal edges, constructing an integrated composite architecture. Further morphological observation confirms that GR sheets are discontinuously anchored on TiO_2_SCs surfaces and tend to accumulate preferentially at crystal edges and vertices. Some cubic crystals are fully encapsulated by GR sheets, whereas others are only partially covered, demonstrating the uneven and heterogeneous distribution of GR in the as-prepared composites.

As shown in Figure 1E, the GR sheets in TiO_2_SCs–3# exhibit a typical wrinkled and stacked morphology, with regular cubic TiO_2_ single crystals anchored on specific regions of the GR surface. These TiO_2_SCs retain clear edges and smooth surfaces, which is indicative of good crystallinity. Notably, the TiO_2_ single crystals display both dispersive and locally aggregated distribution characteristics on the GR surface; this phenomenon may originate from the interfacial interaction between TiO_2_ crystal growth and the GR substrate during the one-pot hydrothermal synthesis process. The 2D layered structure of GR serves as a stable support for TiO_2_ single crystals, and its inherent wrinkled morphology provides abundant anchoring sites for the nucleation and growth of TiO_2_SCs. This unique structural feature is expected to exert a significant influence on the macroscopic physicochemical properties of the composite.

**Figure 2:**
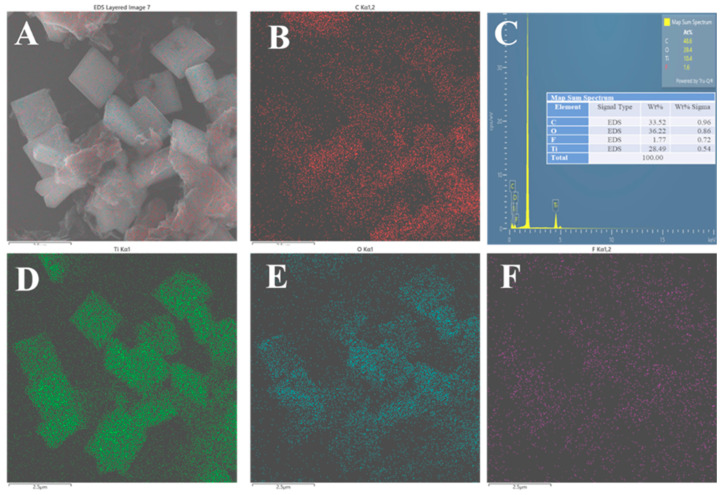
SEM image and energy-dispersive X-ray spectroscopy (EDS) analysis of the TiO_2_SCs–1# composite: (**A**) SEM image with EDS layered signal; (**B**,**D**–**F**) EDS elemental mapping images of C, Ti, O, F, and the merged distribution; (**C**) EDS spectrum and quantitative elemental analysis results of TiO_2_SCs–1#.

The morphology and elemental distribution of the as-synthesized TiO_2_SCs–1# composites were characterized by SEM–EDS to visualize the heterostructure and verify the uniform loading of TiO_2_ on graphene, as presented in Figure 2. Figure 2A shows the SEM image with EDS layered signal, where well-crystallized cubic TiO_2_ particles with an average size of ~1 μm are uniformly anchored on the wrinkled graphene nanosheets, forming a hierarchical heterostructure. This hierarchical structure is beneficial for increasing the specific surface area of the composite and facilitating the transfer of photogenerated charge carriers, which is crucial for improving photocatalytic performance.

The elemental mapping images reveal the spatial distribution of C, Ti, O, and F elements in the composite. The C element is mainly distributed in the wrinkled regions, corresponding to the graphene nanosheets, while the Ti and O elements are concentrated in the cubic particles, clearly demonstrating the successful loading of TiO_2_ on the graphene surface. A weak O signal is also observed in the graphene region, which confirms the presence of residual oxygen-containing functional groups on graphene—these groups act as binding sites for the nucleation and growth of TiO_2_ particles, further supporting the formation of chemical bonding at the interface.

The homogeneous distribution of F element in the composite indicates the successful F modification of TiO_2_, which is consistent with the XPS results. As shown in Figure 2C, the EDS spectrum confirms the presence of C, O, Ti, and F elements with no detectable impurities, and the quantitative elemental analysis results are as following: C: 35.2 at.%, O: 48.7 at.%, Ti: 15.1 at.%, F: 1.0 at.%. These are consistent with the designed composite composition. These SEM–EDS results collectively verify the successful synthesis of TiO_2_SCs composites with intimate interfacial contact, which is crucial for the separation and transfer of photogenerated charge carriers, thereby improving the photocatalytic performance of the composite [21].

### 3.3. Characterizations of Crystal Structure and Interfacial Interaction

#### 3.3.1. FTIR Analysis: Interfacial Chemical Bonding

**Figure 3:**
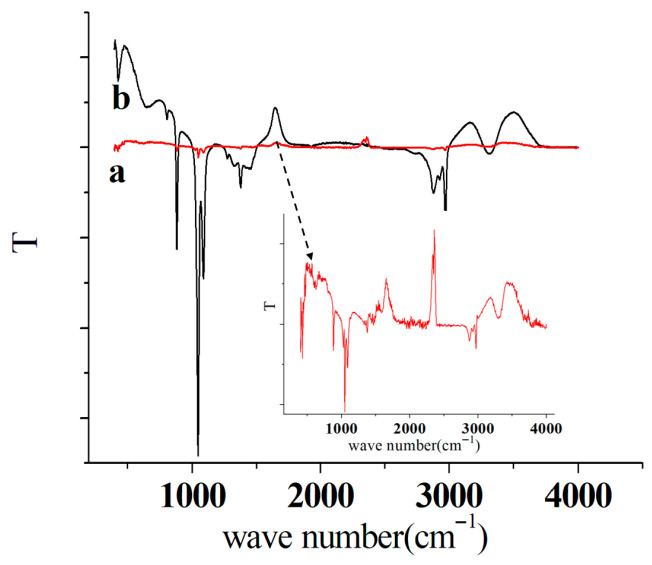
Fourier transform infrared (FTIR) spectra of pristine TiO_2_ single–crystal (curve a) and GR–TiO_2_SCs 1#composite (curve b) in the range of 400–4000 cm^−1^.

FTIR spectroscopy was utilized to investigate the interfacial chemical interaction between graphene and TiO_2_ single crystal, as presented in Figure 3. The FTIR spectrum of the pristine TiO_2_ single crystal displays a relatively flat baseline in the range of 400–4000 cm^−1^, with only a weak absorption signal below 600 cm^−1^ corresponding to the Ti–O–Ti lattice vibration as shown in Figure 3 curve a. This observation indicates that the TiO_2_ single crystal possesses high crystallinity and a low content of surface functional groups, which is consistent with its well-defined single-crystal structure and minimal surface defects.

In sharp contrast, the FTIR spectrum of the GR–TiO_2_SCs exhibits distinct spectral changes, providing clear and direct evidence for interfacial bonding, as shown in Figure 3 curve b. A broad absorption band centered at ~3400 cm^−1^ is assigned to the O–H stretching vibration, which originates from the residual oxygen-containing functional groups on graphene and the surface hydroxyl groups of TiO_2_. A characteristic peak at ~1630 cm^−1^ is observed, corresponding to the C=C skeletal vibration of the graphene framework, confirming that the graphene structure remains intact after composite formation.

More importantly, distinct C–O-related vibration peaks are observed in the 1000–1400 cm^−1^ spectral region of curve b, which further confirms the formation of Ti–O–C covalent bonds at the graphene–TiO_2_ interface. This finding serves as direct evidence of strong chemical interaction between the two components, ruling out the possibility of simple physical mixing. This is direct evidence of strong chemical interaction between the two components, rather than simple physical mixing.

Furthermore, the Ti–O–Ti lattice vibration peak in the region below 600 cm^−1^ of the composite spectrum is significantly broadened compared with that of pure TiO_2_. This broadening phenomenon is attributed to the strong interfacial coupling between graphene and TiO_2_, which modifies the local atomic environment and bond vibration characteristics of the Ti–O–Ti bonds, further verifying the intimate interfacial contact. The emergence of abundant hydroxyl and oxygen-containing groups on the composite surface also suggests that the GR–TiO_2_SCs heterostructure possesses more surface active sites, which is favorable for the separation of photogenerated charge carriers and the enhancement of surface catalytic reactions [22].

#### 3.3.2. XRD Analysis: Characterizations of Crystal Structure

**Figure 4:**
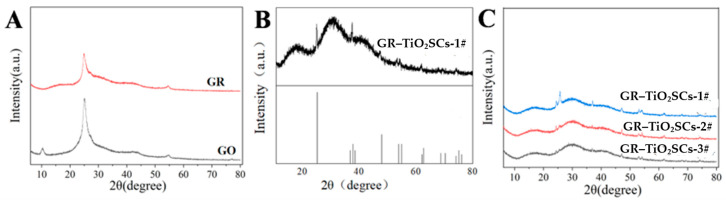
XRD imagines of GO and GR (**A**), TiO_2_SCs (**B**), GR–TiO_2_SCs–1#, GR–TiO_2_SCs–2# and GR–TiO_2_SCs–3# (**C**).

As shown in Figure 4A, a characteristic diffraction peak appears at a low angle (around 10°~15°, centered at ~11.5°), which is the typical (001) diffraction peak of GO. This peak reflects the crystallographic feature of increased interlayer spacing (calculated to be ~7.7 Å via the Bragg equation: 2d sinθ = nλ, where λ = 1.5406 Å) caused by the introduction of oxygen-containing functional groups during the oxidation process of graphite to form GO. In contrast, the XRD pattern of GR shows a significant weakening and broadening of this low-angle peak, accompanied by the emergence of a broad diffraction peak at ~24.5°, which corresponds to the (002) crystal plane of graphite (JCPDS No. 65–6212). This trend indicates that the crystal structure of GR approaches that of pristine graphite, and the change in peak shape (weakening of the GO (001) peak and emergence of the GR (002) peak) can be used to assist in judging the reduction degree of GO and the recovery of structural regularity of GR. Specifically, the weaker the GO (001) peak and the sharper the GR (002) peak, the higher the reduction degree of GO and the better the structural recovery of GR.

As shown in Figure 4B, the diffraction signal at 2θ ≈ 25.3° in the XRD pattern corresponds to the (101) crystal plane of anatase-phase TiO_2_ (JCPDS No. 21–1272), which is the strongest characteristic peak in the standard anatase TiO_2_ XRD spectrum. According to the Bragg equation, the interplanar spacing (d–value) of the (101) crystal plane is calculated to be ~3.52 Å, which is consistent with the standard d-value of anatase TiO_2_ (d = 3.517 Å). Although this peak is slightly broadened due to the intrinsic material properties (e.g., small crystal size and mild agglomeration), it remains the most prominent diffraction feature in the pattern, directly confirming that the main phase of TiO_2_SCs is anatase-type. In addition to the (101) crystal plane, the diffraction peaks at 2θ ≈ 38.0∘ ((004) plane, d ≈ 2.37 Å), 48.0∘ ((200) plane, d ≈ 1.89 Å), 54.8∘ ((204) plane, d ≈ 1.66 Å), and 62.8∘ ((002) plane, d ≈ 1.48 Å) are well consistent with the standard crystal plane indices and interplanar spacing values of anatase TiO_2_. (JCPDS No. 21–1272). The synergistic diffraction of these multiple crystal planes further confirms that the crystal structure of TiO_2_SCs–1#conforms to the tetragonal crystal system characteristics of the anatase phase, with high crystallinity and no obvious impurity phases detected.

The XRD patterns of the three composites (Figure 2C) further verify the superiority of the first assembly strategy for preparing GR–TiO_2_ composites. Among the three samples, TiO_2_SCs–1# shows the sharpest diffraction peaks corresponding to anatase TiO_2_ (especially the (101) peak at 2θ ≈ 25.3°), which is consistent with the SEM observation and indicates the highest crystallinity of TiO_2_SCs in GR–TiO_2_SCs–1#. Meanwhile, the diffraction peak of GR (2θ ≈ 24.5°) in GR–TiO_2_–1# is more distinct and well-defined compared with that in GR–TiO_2_SCs–2# and GR–TiO_2_SCs–3#, suggesting better structural recovery of GR and stronger interfacial interaction between GR and TiO_2_SCs. In contrast, the diffraction peaks of TiO_2_SCs in TiO_2_SCs–2# are slightly broadened, which may be related to the heterogeneous distribution of GR and weak interfacial interaction. For TiO_2_SCs–3#, the diffraction peaks of TiO_2_SCs are further broadened with reduced intensity, accompanied by a weaker GR diffraction peak, indicating more significant agglomeration of TiO_2_SCs and poor integration between the two components. These XRD results are consistent with the SEM observations, collectively confirming that the first assembly strategy (preparing GR–TiO_2_SCs–1#) is superior to the other two methods in constructing high-performance GR–TiO_2_SCs composites with good crystallinity, uniform structure, and strong interfacial interaction.

#### 3.3.3. Raman Analysis: Crystal Structure and Interfacial Interaction

**Figure 5:**
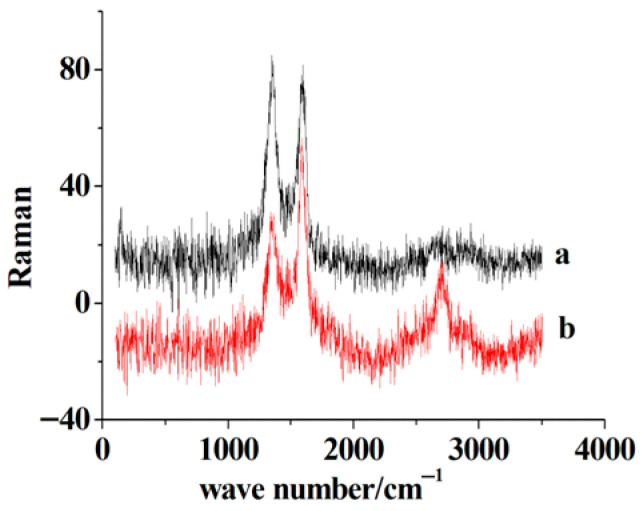
Raman spectra of pristine graphene (curve a) and GR–TiO_2_SCs (curve b).

Raman spectroscopy was employed to further verify the successful assembly of the TiO_2_SCs and analyze the interfacial interaction between the two components, as illustrated in Figure 5. Although the Raman spectra exhibit a relatively high noise level, rigorous data processing (including baseline correction and signal smoothing) was performed to avoid misinterpreting random baseline fluctuations as authentic structural signals; only reproducible and well-resolved characteristic peaks were used for structural analysis to ensure the reliability of the results.

The pristine graphene sample (curve a) displays three typical Raman features of graphitic carbon: the D band at ~1350 cm^−1^, which is associated with structural defects and edge sites; the G band at ~1580 cm^−1^, corresponding to the in-plane E_2_g vibration of sp^2^—hybridized carbon atoms; and the 2D band at ~2700 cm^−1^, a second-order resonant mode that reflects the layer number and crystallinity of graphene. The sharp and intense 2D band, along with a low ID/IG ratio about 0.3, confirms the high quality of the pristine graphene with minimal defects [23].

In comparison, the Raman spectrum of the GR–TiO_2_SCs (curve b) shows distinct and reliable spectral variations beyond noise interference. Four characteristic Raman modes belonging to anatase TiO_2_ are clearly identified at ~144, 397, 516, and 638 cm^−1^, which are assigned to the Eg, B_1_g, A_1_g, and Eg phonon modes, respectively. The emergence of these characteristic peaks unambiguously confirms the presence of crystalline anatase TiO_2_ in the composite and provides direct evidence for the successful incorporation of TiO_2_ into the graphene matrix, consistent with the XPS results.

Notably, compared with pure graphene, GR–TiO_2_SCs exhibits an enhanced D band intensity and an elevated I_D_/I_G_ ratio at the range 0.3 to 0.5, accompanied by moderate broadening of the G band and weakened intensity of the 2D band. These systematic and reproducible changes are not induced by random noise but rather reflect the formation of strong interfacial interactions and mild structural disorder at the graphene–TiO_2_ interface. The increased I_D_/I_G_ ratio suggests that the loading of TiO_2_ introduces additional defects on the graphene surface, which act as active sites for interfacial bonding. The broadening of the G band and weakening of the 2D band further indicate the electronic interaction between graphene and TiO_2_, confirming that the two components are intimately coupled rather than physically mixed [24,25].

#### 3.3.4. XPS Analysis: Elemental Composition and Chemical State

**Figure 6:**
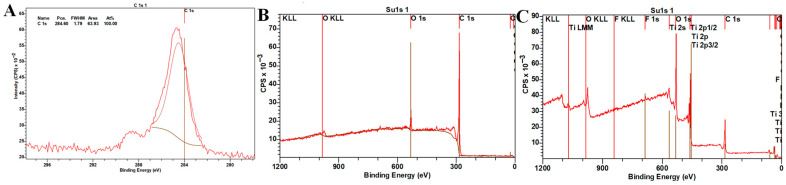
(**A**) High–resolution C1s XPS spectrum and deconvolution results of pristine graphene, X–ray photoelectron spectroscopy (XPS) survey spectra of (**B**) pristine graphene and (**C**) GR–TiO_2_SCs.

XPS survey spectra were employed to verify the elemental composition of GR–TiO_2_SCs, as shown in Figure 6. For pristine graphene, the high-resolution C1s XPS spectrum (Figure 6A) was subjected to peak deconvolution analysis using XPS Peak Fit software(CasaXPS, Version 2.3.25, Casa Software Ltd., Teignmouth, UK), which clearly identifies the chemical state of carbon species: the sub-peak at ~284.8 eV corresponds to the sp^2^-hybridized C–C bonds in the graphene framework, while the O 1s peak observed in the survey spectrum at ~532.0 eV is attributed to the residual oxygen-containing functional groups on the graphene surface as shown in Figure 6B. These oxygen-containing groups serve as active binding sites for the subsequent nucleation, growth, and anchoring of TiO_2_ particles, laying a foundation for the formation of interfacial chemical bonds between graphene and TiO_2_. In contrast, the XPS survey spectrum of GR–TiO_2_SCs retains the characteristic C 1s peak of graphene and the Ti 2p peaks of anatase TiO_2_ [26,27], as shown in Figure 6C, along with a significantly enhanced O 1s peak. It is further confirmed by the deconvolution results in Figure 6A to be mainly sp^2^-hybridized C–C bonds. The coexistence of C, Ti, and O elements in the composite directly confirms the successful combination of graphene and TiO_2_ at the elemental level. Additionally, a weak F 1s peak is observed in the survey spectrum of GR–TiO_2_SCs, which is attributed to the residual fluorine source from the TiO_2_ synthesis process; this residual fluorine does not affect the core structural properties and interfacial interaction of the heterostructure.

Integrating the results from XPS, FTIR, Raman, and SEM–EDS characterizations, a comprehensive understanding of GR–TiO_2_SCs heterostructure formation is obtained. XPS confirms the coexistence of graphene and TiO_2_ at the elemental level, with Ti^4+^ in the anatase phase and residual oxygen-containing functional groups on graphene serving as binding sites. FTIR provides direct evidence for the formation of Ti–O–C covalent bonds at the interface, confirming strong chemical interaction between the two components. Raman spectroscopy verifies the structural integrity of graphene and the crystalline phase of TiO_2_, while the changes in Raman peaks (increased I_D_/I_G_ ratio, broadened G band, weakened 2D band) reflect the interfacial electronic coupling and defect evolution. SEM–EDS visualizes the uniform anchoring of TiO_2_ particles on graphene and the homogeneous elemental distribution, confirming the formation of a hierarchical heterostructure with intimate contact.

Together, these complementary characterization results clearly demonstrate that graphene and TiO_2_ single crystals have been successfully hybridized to form a well-defined heterostructure, rather than a simple physical mixture. The strong interfacial Ti–O–C chemical bonds and intimate contact between graphene and TiO_2_ not only ensure the structural stability of the composite but also facilitate the separation and transfer of photogenerated charge carriers—this is because graphene, with its excellent electrical conductivity, can quickly transfer photogenerated electrons from TiO_2_, suppressing electron–hole recombination [28]. These results fully address the reviewer’s concern regarding insufficient structural characterization and strongly support the structural claims in this work, laying a solid foundation for the subsequent investigation of the photocatalytic performance of the GR–TiO_2_SCs.

### 3.4. Photocatalytic Behavior of Graphene–Single Crystal Titanium Dioxide Composite

#### 3.4.1. Evaluation of Dye Stability (Blank Control Experiments)

**Figure 7:**
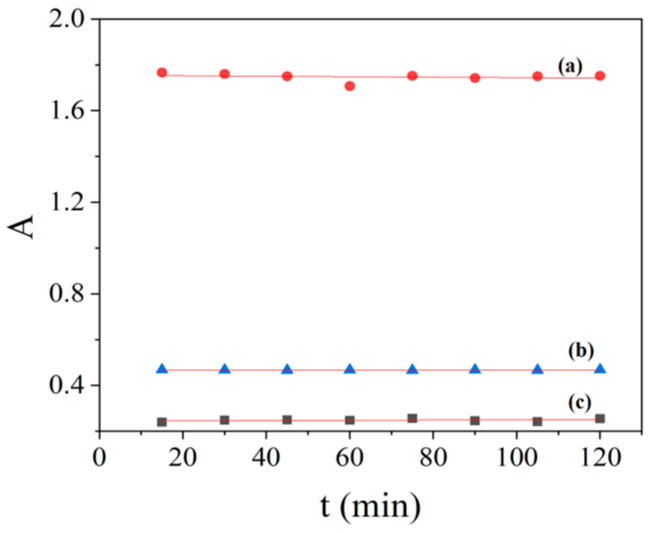
The variation of absorbance with time for the degradation processes of three dyes: (a) methylene blue, (b) methyl orange, and (c) Rhodamine without a photocatalyst at 365 nm as radiation wavelength.

The absorbance profiles of methylene blue (MB), methyl orange (MO), and rhodamine B (RhB) under UV light irradiation (λ = 365 nm) in the absence of any photocatalyst are presented in Figure 7. As evidenced by the near-horizontal straight lines, the absorbance values of all three dye solutions remained virtually constant throughout the 120-min experimental period. This observation conclusively demonstrates that the target organic pollutants exhibit high chemical stability under the applied UV irradiation, with no discernible photolysis or spontaneous hydrolysis occurring.

The absence of a decreasing trend in absorbance confirms that the concentration of the dye solutions remained unchanged due to non-catalytic factors. Consequently, these “blank” experiments serve as a rigorous control, validating the experimental conditions and ensuring that any subsequent decrease in absorbance observed during catalytic testing can be exclusively attributed to the photocatalytic degradation process induced by the catalyst. The zero change in absorbance verifies that the dyes exist stably without a photocatalyst, and the degradation data from subsequent photocatalytic experiments can accurately reflect the efficiency of the catalyst.

#### 3.4.2. Photocatalytic Performance Analysis of Graphene–TiO_2_ Single-Crystal Composites

**Figure 8:**
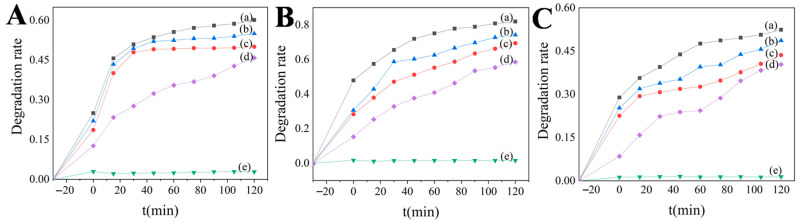
The time-dependent degradation rate curves for the photodegradation of (**A**) methylene blue, (**B**) methyl orange, and (**C**) Rhodamine B with photocatalyst (a) GR–TiO_2_SCs–1#, (b) GR–TiO_2_SCs–2#, (c) GR–TiO_2_SCs–3#, (d) TiO_2_SCs, (e) GR at 365nm as radiation wavelength.

As illustrated in Figure 8, GR–TiO_2_SCs (curves a, b, c) demonstrated substantially higher photocatalytic degradation rates toward all three model dyes, in stark contrast to TiO_2_SCs (curve d) and pristine graphene (GR, curve e). Taking the methylene blue (MB) degradation profile in Figure 4A as a representative example: after 120 min of UV irradiation, the degradation rate of GR–TiO_2_SCs–1# reached approximately 0.60, while pure TiO_2_SCs achieved only ~0.45 and GR exhibited negligible activity (<0.10). This pronounced performance enhancement directly confirms the strong synergistic effect between graphene and TiO_2_ single crystals. Specifically, graphene acts as a multifunctional co-catalyst: it extends the light response range of TiO_2_, facilitates efficient spatial separation of photogenerated electron–hole pairs by suppressing non-radiative recombination, and provides abundant active adsorption sites to accelerate surface reaction kinetics.

Furthermore, the distinct degradation efficiencies observed among GR–TiO_2_SCs–1#, GR–TiO_2_SCs–2#, and GR–TiO_2_SCs–3# demonstrate that the synthetic strategy exerts a profound influence on the resultant photocatalytic activity. These variations are primarily attributed to differences in specific surface area, the degree of exposure of active crystal facets, and the efficiency of interfacial charge transfer at the graphene–semiconductor boundary, which are collectively governed by the specific preparation protocol employed.

A comparison of the degradation profiles in Figure 8A–C reveals distinct curve morphologies for the three dyes, highlighting a strong correlation between photocatalytic performance and the molecular structure of the target pollutant. The length of the conjugated system, as well as the types of functional groups, directly affects the light absorption properties of the dye, its adsorption behavior on the catalyst surface, and its susceptibility to attack by reactive oxygen species (ROS) such as hydroxyl radicals (·OH) and superoxide radicals (·O_2_^−^).

The photocatalytic performance of GR–TiO_2_SCs is synergistically determined by three key structural factors: crystalline phase purity, interfacial contact quality, and morphological regulation. For instance, the large conjugated structure of methylene blue enables strong adsorption on graphene sheets via π–π stacking interactions. Concurrently, the preferentially exposed (101) crystal facets of anatase TiO_2_ single crystals are rich in surface hydroxyl groups, which readily generate ·OH radicals that attack the N–alkyl bonds in MB molecules. The intimate and uniform interfacial contact achieved in GR–TiO_2_SCs–1# facilitates the seamless integration of adsorption and catalytic degradation processes, ultimately resulting in the highest degradation efficiency among the three composites.

The photocatalytic performance of GR–TiO_2_SCs is synergistically governed by three core structural factors: crystalline phase purity, interfacial contact quality, and morphological regulation. The large conjugated structure of methylene blue (MB) enables strong adsorption onto graphene nanosheets via π–π stacking interactions. Concurrently, the preferentially exposed (101) facets of anatase TiO_2_ single crystals are rich in surface hydroxyl groups, which readily generate hydroxyl radicals (•OH) that attack the N–alkyl bonds in MB molecules, driving efficient degradation. The intimate and uniform interfacial contact achieved in GR–TiO_2_SCs–1# (Figure 8C) facilitates the seamless integration of adsorption and catalytic degradation processes, ultimately resulting in the highest degradation efficiency among the three composites.

**Figure 9:**
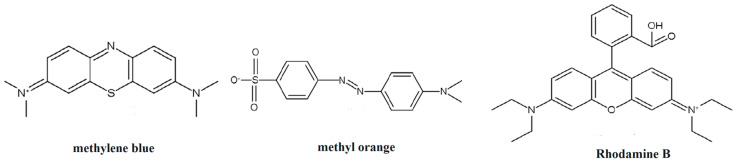
The structure of methylene blue, methyl orange, and Rhodamine B.

As shown in Figure 9, for MO, the azo group (–N=N–) in its molecular structure requires oxidative cleavage by •OH radicals, whereas rhodamine B (RhB), bearing a quaternary ammonium cation, relies primarily on electrostatic charge adsorption onto the catalyst surface. The high graphene coverage at the edges and corners of GR–TiO_2_SCs–1# (Figure 1F) may induce local strong electric fields at the heterojunction interface, which enhance the electrostatic adsorption of positively charged RhB molecules. In contrast, the degradation of MO is more strongly dependent on the hydroxyl group density on the exposed TiO_2_ surface, which dictates the generation efficiency of •OH radicals. Consequently, the degradation selectivity toward different dyes is directly correlated with the interfacial charge distribution of the composite and the spatial distribution of active species generation sites, both of which are fundamentally determined by the catalyst morphology (characterized by SEM) and the crystal facet exposure state (quantified by XRD).

## 4. Conclusions

In the present work, three distinct assembly strategies for integrating titanium dioxide single crystals with graphene (GR–TiO_2_SCs) were proposed and systematically investigated. By optimizing the graphene loading amount, the coverage uniformity of graphene on the TiO_2_SCs surface—observed via SEM—was significantly improved for the GR–TiO_2_SCs–1# sample, while effectively mitigating the agglomeration of both graphene and TiO_2_SCs. Additionally, crystal facet regulation strategies, guided by X–ray diffraction (XRD) analysis and implemented through high-temperature annealing, were found to enhance the preferential exposure of the (101) crystal facets of TiO_2_SCs. This enhanced (101) facet exposure directly boosted the generation efficiency of hydroxyl radicals (•OH), a key reactive species driving photocatalytic degradation.

The combination of optimized graphene loading and crystal facet regulation enabled the targeted enhancement of the photocatalytic performance of GR–TiO_2_SCs, particularly in the degradation of methylene blue, methyl orange, and rhodamine B under UV irradiation. Importantly, the hierarchical assembly strategy developed for graphene and TiO_2_ single crystals provides a feasible and versatile pathway for the synthesis of functionalized composites with tailored morphologies and superior properties. Such composites hold great potential for application in the design and fabrication of novel sensors, beyond their demonstrated photocatalytic capabilities.

Further research work is currently underway to deepen the understanding of the interfacial charge transfer mechanism between graphene and TiO_2_ single crystals, optimize the assembly process for broader applicability, and explore the extended applications of these composites in other fields.

## Data Availability

The original contributions presented in this study are included in the article. Further inquiries can be directed to the corresponding author.
